# Chondrosarcoma presenting as dyspnea in a 19-year-old man: a case report

**DOI:** 10.1186/1752-1947-5-150

**Published:** 2011-04-15

**Authors:** Rajasekharan Chandrasekharan, Mithun Chalakarayil Bhagavaldas, Ashish Jacob Mathew

**Affiliations:** 1 Department of Internal Medicine, Medical College Hospital, Trivandrum, Kerala, India

## Abstract

**Introduction:**

Acute pulmonary embolism has varied presentations ranging from asymptomatic, incidentally discovered emboli to massive embolism, causing immediate death. Tumor embolism is a rare but unique complication of malignancies. This uncommon catastrophe of a malignant tumor in a young patient, culminating as a pulmonary embolism, is being reported for the first time.

**Case presentation:**

A 19-year-old Asian man presented to the emergency service at our hospital with acute onset dyspnea. His clinical examination led to the suspicion of an acute pulmonary embolism with a lower lumbosacral radiculopathy. A magnetic resonance imaging scan of the pelvis demonstrated a chondrosarcoma arising from the right iliac wing, eroding into the common iliac vein and creeping up the inferior vena cava to lodge in the pulmonary artery, thus producing a saddle embolus.

**Conclusion:**

The importance of exploring for malignancies in the event of an idiopathic pulmonary embolism is highlighted. Early detection of such malignancies can substantially affect the outcome in young patients.

## Introduction

Detached thrombi or tumour may be the cause for massive pulmonary embolism in patients with malignancies. Identification of the type of pulmonary embolism is cardinal as the treatment and prognosis vary considerably. We report an unusual presentation of a tumour embolism in a young man, who succumbed to the disease.

## Case report

A 19-year-old Asian man, was admitted to the emergency services (ES) at our hospital with acute onset of severe breathlessness following a short duration of dry cough, without any associated fever, chest pain or hemoptysis. He also had lower-back pain and right lower limb weakness with paresthesia, which was attributed to an injury sustained while playing football. He was prescribed analgesics and advised by an orthopedic surgeon to take bed rest for his lower limb ailment. His lower-back radiograph was normal. On examination at the ES, he had tachypnea and tachycardia with elevated jugular venous pressure and was normotensive with no pallor or pedal edema. His cardiovascular system examination revealed a loud second heart sound and a right ventricular third heart sound. His respiratory system examination was normal. The examination of his nervous system was suggestive of a right lower lumbosacral (L3-S1) radiculopathy. A moderate, tender hepatomegaly was detected in abdominal palpation. His oxygen saturation in the ES was 92 percent at room air temperature.

**Figure 1:**
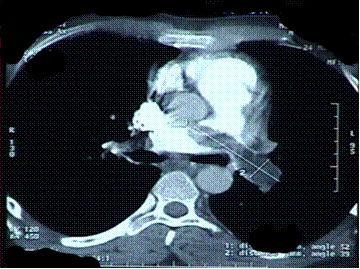
**High-resolution computed tomographic image of the patient's chest showing the saddle embolus**.

Initial blood investigations revealed leukocytosis with neutrophilia and normal liver and renal function. An acid base analysis showed compensated respiratory acidosis. His chest radiograph revealed a wedge-shaped opacity in the right midzone with dilated main pulmonary artery and focal oligemia in the right lower zone, which may have been suggestive of a pulmonary embolism. High-resolution computed tomographic (HRCT) imaging showed an acute, large, saddle embolus completely filling the left pulmonary artery and partly occluding the right pulmonary artery, as well as multiple, detached peripheral pulmonary artery thrombi in both the upper and right lower lobes (Figure 1). Emergency echocardiography was performed, which revealed a large thrombus at the main pulmonary artery bifurcation and extending into the right and left pulmonary arteries. The right atrium and main pulmonary artery were dilated.

**Figure 2:**
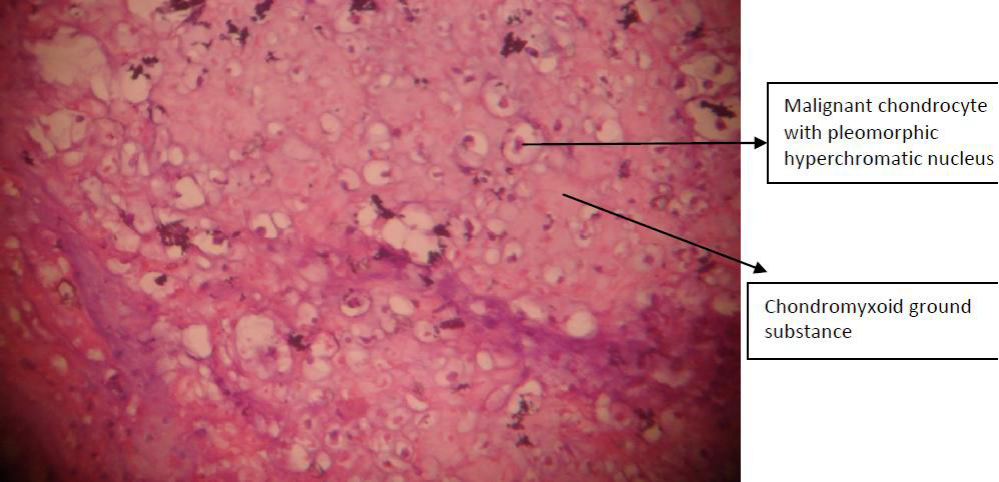
**Pulmonary embolectomy specimen showing malignant chondrocytes**.

Considering the short duration of symptoms and the massive size of the thrombus, thrombolysis was tried with streptokinase followed by unfractionated heparin infusion. On the second day of admission, the patient developed hemoptysis and signs of deep vein thrombosis in both the lower limbs, and his oxygen saturation dropped to 86% at room air temperature. A Doppler ultrasound scan of both lower limbs demonstrated an extensive acute thrombus involving the peroneal vein, distal femoral vein, popliteal vein, the entire segment of the external iliac vein, the common iliac vein on the right side and the inferior vena cava. As the thrombus was refractory to thrombolysis therapy, a surgical embolectomy of the pulmonary embolism was attempted, the histopathological examination of which revealed a chondrosarcoma (Figure 2).

**Figure 3:**
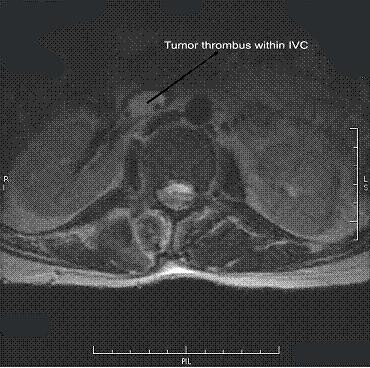
**Magnetic resonance imaging scan showing tumor embolus within the inferior vena cava**.

**Figure 4:**
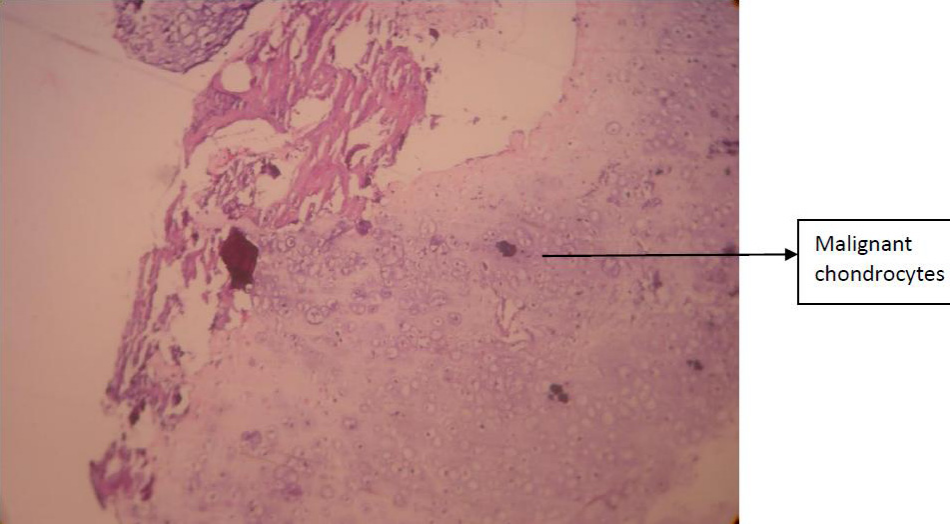
**Specimen from the iliac bone showing malignant chondrocytes**.

The postoperative period was uneventful, and his symptoms subsided gradually. Because we suspected a tumor embolism, his lower-back ache and paresthesia were evaluated in detail. A magnetic resonance imaging (MRI) scan of his spine and pelvis showed a large, destructive mass lesion arising from the right iliac wing with permeative lytic areas involving the right iliac vein and a tumor embolus within the inferior vena cava (Figure 3). There were metastases involving the fourth and fifth lumbar and first sacral vertebral bodies as well as pedicles on the right side. An open biopsy was taken from the iliac tumor, which confirmed the diagnosis (Figure 4). We initiated palliative chemotherapy for the patient. As there was no conclusive evidence of thromboembolism, anticoagulation was deferred.

## Discussion

Malignancy is one of the well-known risk factors for pulmonary embolism. Rarely, embolism of the tumor tissue itself or tumor cells can occlude the main pulmonary artery or its large branches, causing dyspnea. This is called tumor embolism and is exceedingly difficult to recognize before death. Autopsy series have estimated the incidence of pulmonary tumor embolism to be between 3% and 26% among patients with solid tumors [1,2]. The majority of reported cases are in association with breast, stomach or lung carcinomas [3]. Chondrosarcomas, the third most common primary malignancy of the bone, usually grow slowly and metastasize [4]. In some cases, however, this course is altered dramatically by a long-recognized feature of this tumor: intravascular invasion.

Very few case reports have confirmed the association of tumor embolism and chondrosarcoma [5-7]. In the case reported here, a pelvic chondrosarcoma was responsible for the thrombus. The unusual feature of this tumor was its transvenous spread involving the pelvic and retroperitoneal veins all the way to the inferior vena cava (Figure 3) and causing subsequent tumor embolization. MRI and Doppler ultrasound scans together could detect the invasion. No report of a major case series of chondrosarcoma has discussed this complication.

A good proportion of patients with tumor embolisms already have widespread metastatic disease at the time of presentation. Hence, there is a paucity of prospective trials of chemotherapy in these patients. The treatment options available in such situations are surgical embolectomy and treatment of the primary tumor, which includes wide *en bloc *excision, radiotherapy, palliative care and antiangiogenesis combined with chemotherapy [4,8]. Few cases of successful chemotherapy of tumor embolism have been described in the literature [9,10]. The most important decision involving a patient suspected of having a tumor embolism is how aggressively to pursue the diagnosis. An early confirmation of the presence of tumor embolism may avoid unnecessary anticoagulation and thrombolytic therapy, which are absolute indications in cases of thromboembolism. There are also chances of complete remission of the tumor if adequate chemotherapy is initiated well in advance. The patient reported here showed symptomatic improvement after surgery. However, the tumor had already caused widespread damage. Hence, radiotherapy had to be initiated, followed by palliation.

## Conclusion

Occurrence of an acute pulmonary embolism in a cancer patient should always prime the clinician to search for tumor thrombosis. The refractoriness of the thrombus to thrombolysis therapy, as in the reported case, is yet another pointer to tumor embolism. Whenever chondrosarcoma of the pelvis is suspected in a young patient, the possibility of extensive venous extension should be considered and appropriate diagnostic tests should be performed early to avoid unnecessary anticoagulation and initiate appropriate chemotherapy.

## Consent

Written informed consent was obtained from the patient's father for publication of this case report and accompanying images. A copy of the written consent is available for review by the Editor-in-Chief of this journal.

## Competing interests

The authors declare that they have no competing interests.

## Authors' contributions

RC was the primary clinician and prepared the manuscript. MCB was the resident in charge of the patient. AJM was the resident in the unit and prepared the manuscript. All the authors have read and approved the final manuscript.
